# Supplementary material for: Integration of elemental imaging and spatial transcriptomic profiling for proof-of-concept metals-based pathway analysis of colon tumor microenvironment
Source: Metallomics. 2025 Oct 3;17(10):mfaf034. doi: 10.1093/mtomcs/mfaf034 (PMC12569514; doi:10.1093/mtomcs/mfaf034)
Supplement: mfaf034_Supplemental_Files [file mfaf034_supplemental_files.zip › spatial_multimodal_colon_revisions_metallomics_supplemental.docx]

**Supplementary Materials**

**Supplementary Figures**


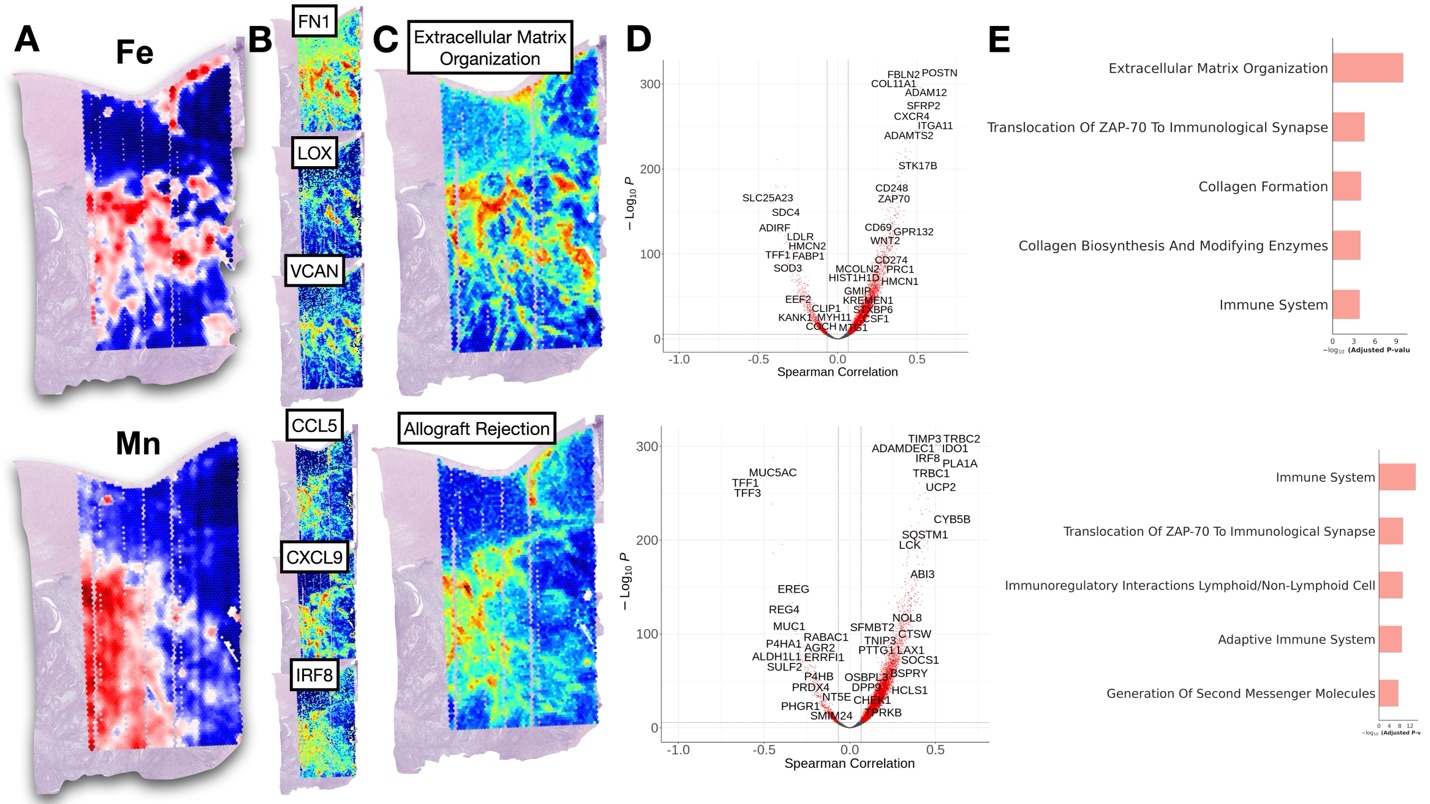


**Figure S1: Additional Metals-Based Pathway Findings Based on Spatial Transcriptomics Correlation with Fe and Mn Distribution: A)** Fe and Mn Gi* hotspot statistics, **B)** Visualization of spatial distribution of Fe-related (FN1, LOX, VCAN) and Mn-related (CCL5, CXCL9, IRF8) gene expression demonstrating association with respective elements, **C)** Visualization of Extracellular Matrix Organization and Allograft Rejection (reflects anti-cancer immune response) through aggregating gene expression across pathway compared to a background distribution, again found to be associated with respective elements, **D)** Volcano plot mapping spearman correlation between 18074 genes and Gi* statistics for respective metals on x-axis and -log10(p-values) on y-axis– p-value and correlation cutoffs were set at 0.05/18074; **E)** Pathways associated with respective elements (Reactome pathways)

**
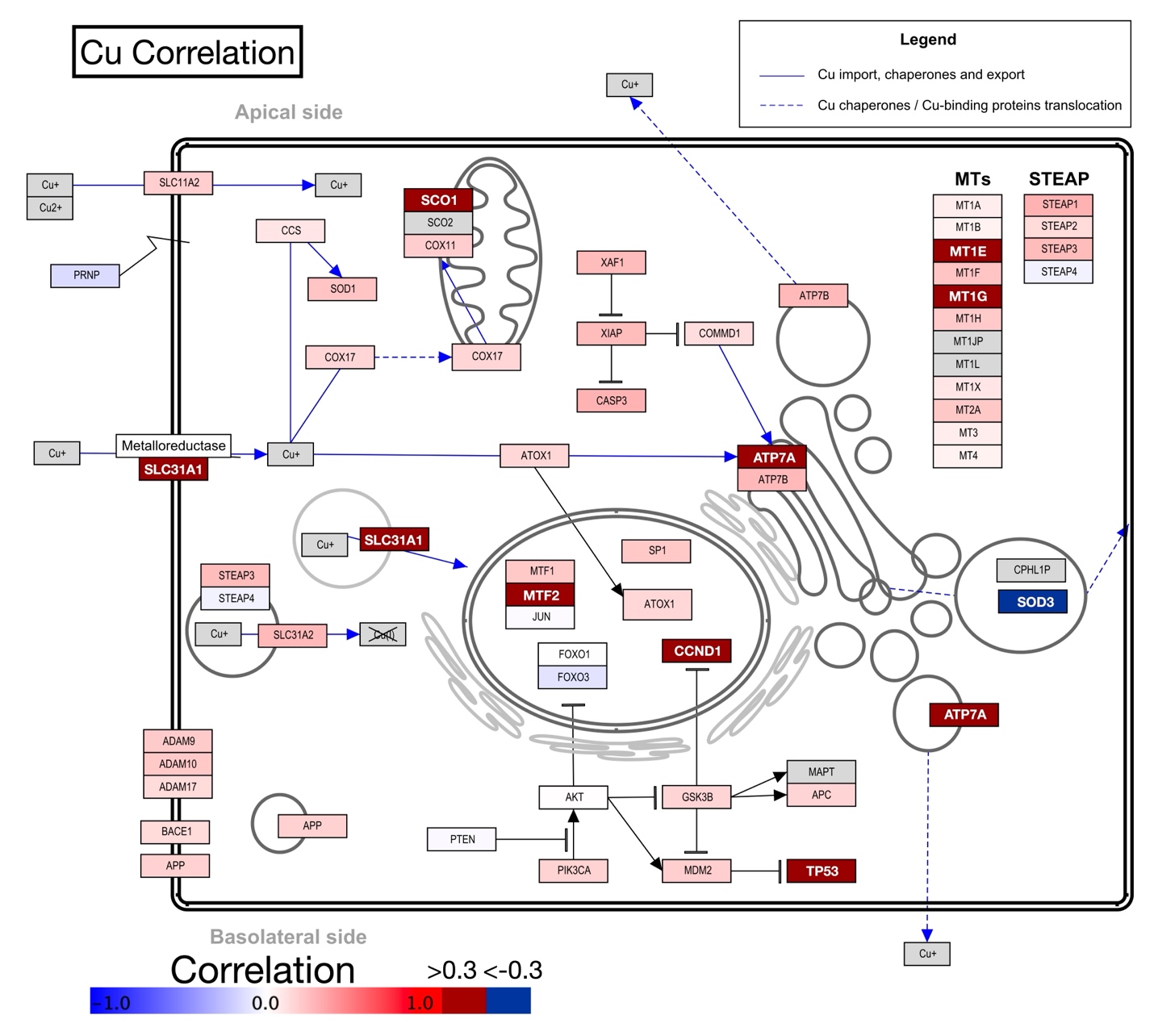
**

**Figure S2: Visualization of Cu Gene Expression Correlation Results Overlaid on Cu Homeostasis Pathway Diagram:** Color of each gene reflects positive (red) and negative (blue) correlations with Cu distribution; correlations with magnitude exceeding 0.3 are denoted using dark red/blue

**
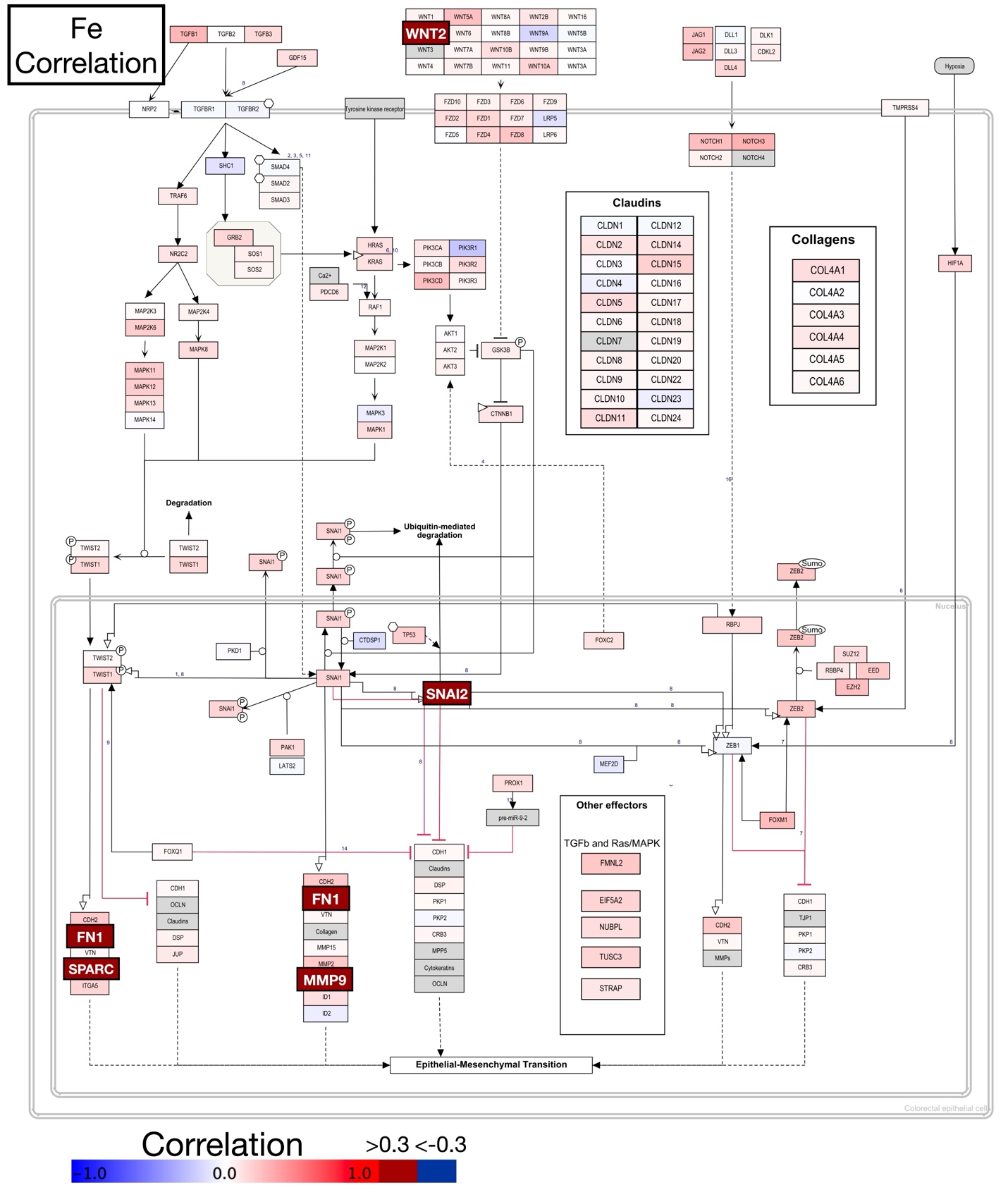
**

**Figure S3: Visualization of Fe Gene Expression Correlation Results Overlaid on Epithelial-to-Mesenchymal Pathway Diagram:** Color of each gene reflects positive (red) and negative (blue) correlations with Cu distribution; correlations with magnitude exceeding 0.3 are denoted using dark red/blue

**
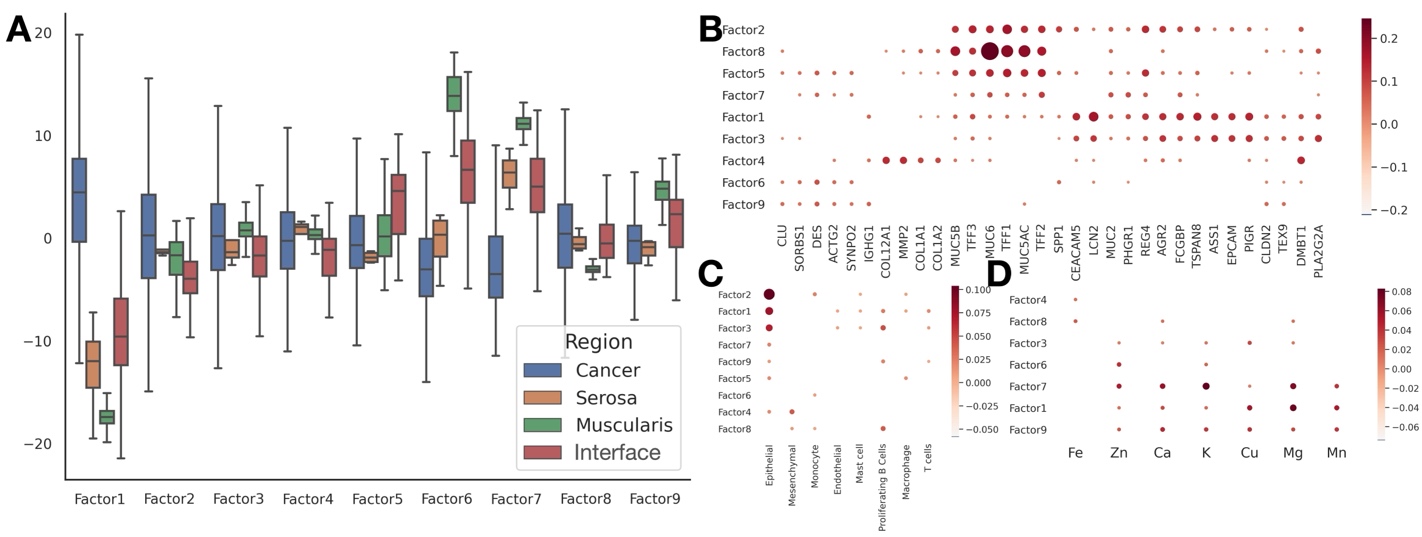
**

**Figure S4: Further Factor Analysis Associations, broken down by: A)** Tissue architecture (average loading), **B)** Gene expression (factor coefficients), **C)** cell type (factor coefficients), **D)** element (factor coefficients)

**
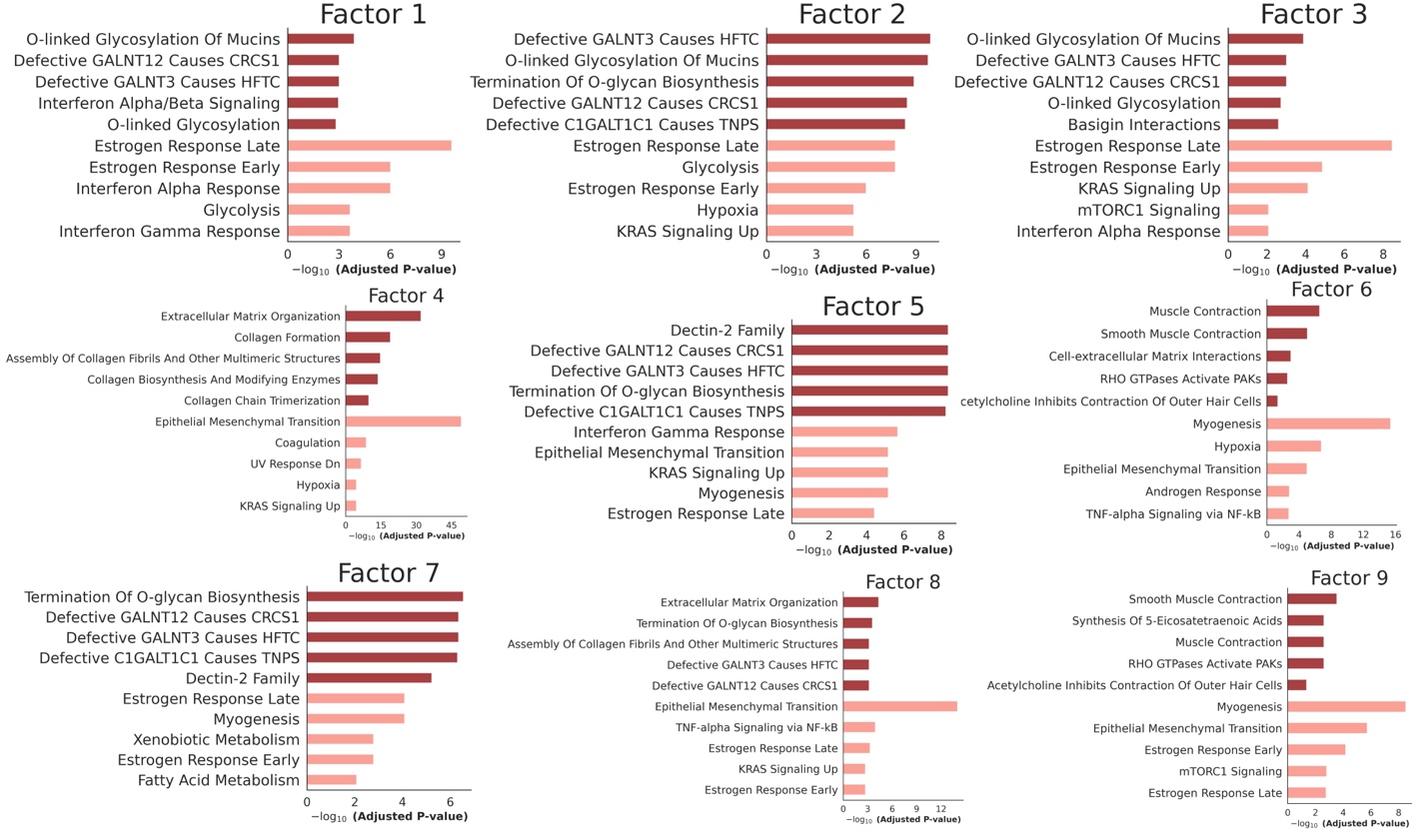
**

**Figure S5: Association of Spatial Factors with Biological Pathways** from Reactome (Dark Red) and MsigDB Cancer Hallmarks (Salmon)

**
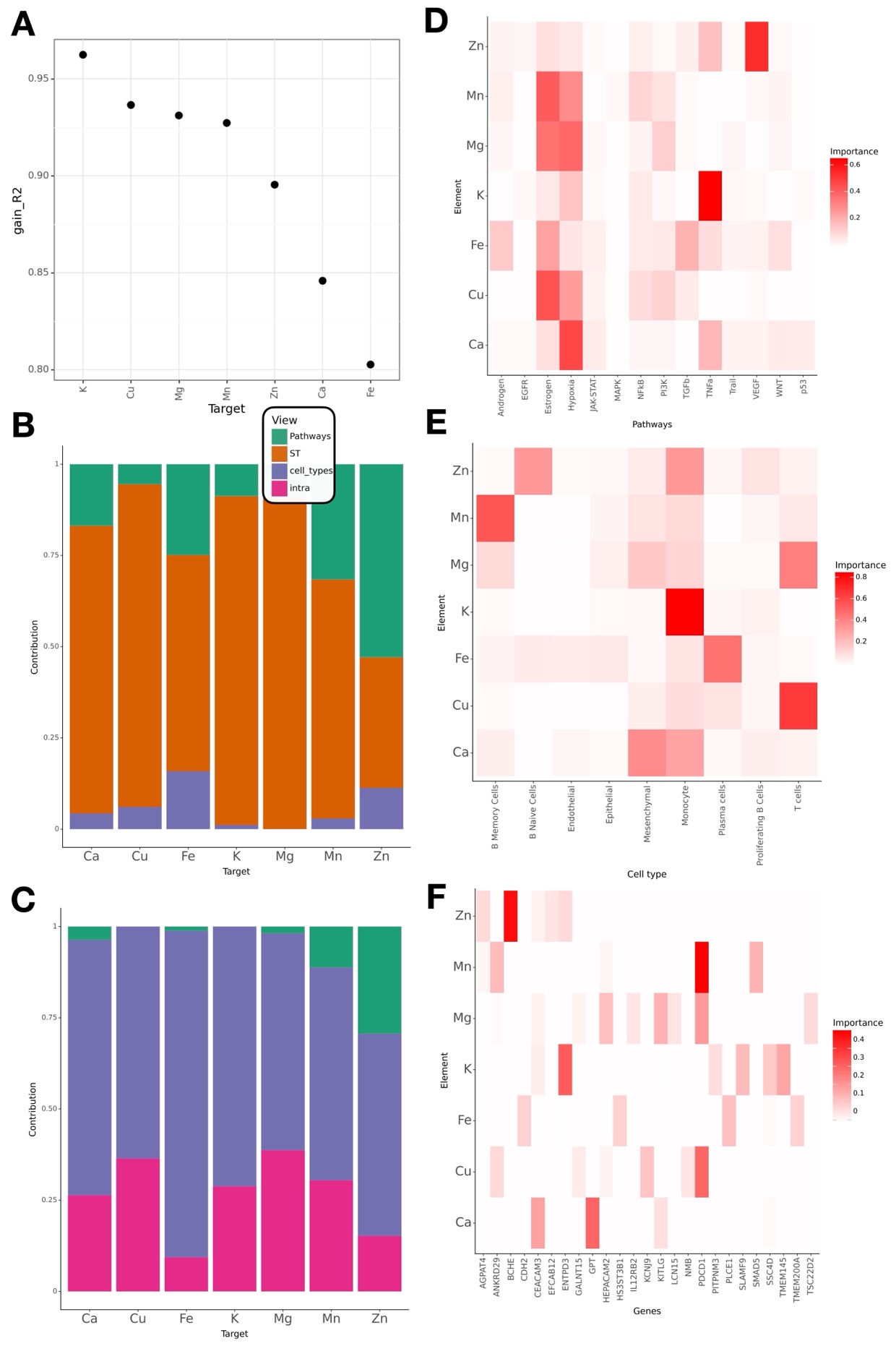
**

**Figure S6: Elemental Distribution and Variance Explained via MISTy: A)** Ability to predict various elements by ST data types alone via R², **B)** Breakdown of R² by data type, **C)** Breakdown of R² by data type when including other elements, **D-F)** Spatially informative features, specific to each element, broken down by **D)** Pathway, **E)** Cell type, **F)** Gene

**
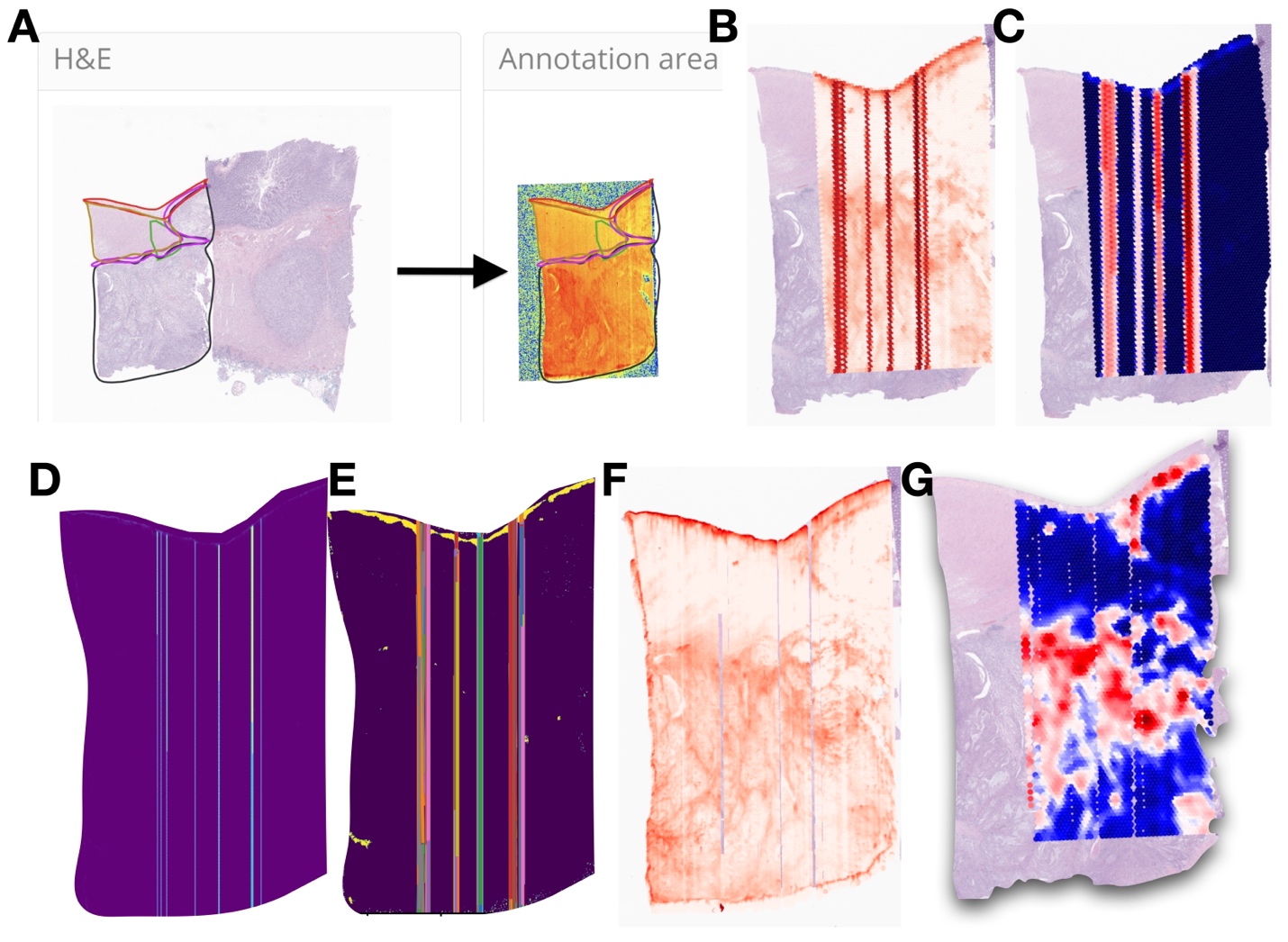
**

**Figure S7: Preprocessing Workflow: A)** Elemental maps are co-registered to ST-associated H&E WSI via TRACE, **B)** Vertical strip artifacts in Fe are **C)** confounding hotspot analysis and they are removed through **D)** anisotropic diffusion filtering, followed by **E)** hough transform for line detection. **F)** Elemental image after removing strip artifact. **G)** Removal of edge bleeding/washout artifact through binary erosion, aggregation of elemental abundance to ST spot level, and derivation of Gi* hotspot statistics

**Supplementary Tables**

**Table S1:** Association of elemental abundance with architecture (one versus rest), includes average Gi* spot-level statistics by architecture and marginal mean calculated through regression modeling along with statistical significance (see TableS1.xlsx)

**Table S2:** Association of elemental abundance with pairwise relative differences between architectures, includes marginal mean comparisons calculated through regression modeling along with statistical significance (see TableS2.xlsx)

**Table S3:** Association of elemental abundance with cell-type, includes spearman correlations and p-values (see TableS3.xlsx)

**Table S4:** Gene expression metal correlation analysis results, includes spearman correlation, p-values and Bonferroni-adjusted p-values by Element (see TableS4.xlsx)

**Table S5:** Pathway analysis findings (Reactome, Hallmarks) for top 150 genes tied to higher elemental abundance, ranked by z-statistic (see TableS5.xlsx)

**Table S6:** Pathway analysis findings (Reactome, Hallmarks) for top 150 genes tied to lower elemental abundance, ranked by z-statistic (see TableS6.xlsx)

**Table S7:** Pathway analysis findings (Reactome, Hallmarks) for top 150 genes tied to higher and lower elemental abundance, ranked by magnitude of z-statistic (see TableS7.xlsx)

**Table S8:** Composition of spatial factors, MEFISTO factor analysis weights by top 10 genes, cell-types and elements (see TableS8.xlsx)

**Table S9:** Association of spatial factors with architecture (one versus rest), includes averaged factor loadings by architecture and marginal mean calculated through regression modeling along with statistical significance (see TableS9.xlsx)

**Table S10:** Association of spatial factors with pairwise relative differences between architectures, includes marginal mean comparisons calculated through regression modeling along with statistical significance (see TableS10.xlsx)

**Table S11:** MISTy performance statistics and feature importances, includes metal abundance prediction performance broken down by data type (cell type information, spatial transcriptomics, pathways, metals/intra-view) with and without inclusion of other metals in model and Gini-index of features by data type (see TableS11.xlsx)
