# Supplementary figures and images for: Integration of elemental imaging and spatial transcriptomic profiling for proof-of-concept metals-based pathway analysis of colon tumor microenvironment
Source: Metallomics. 2025 Oct 3;17(10):mfaf034. doi: 10.1093/mtomcs/mfaf034 (PMC12569514; doi:10.1093/mtomcs/mfaf034)

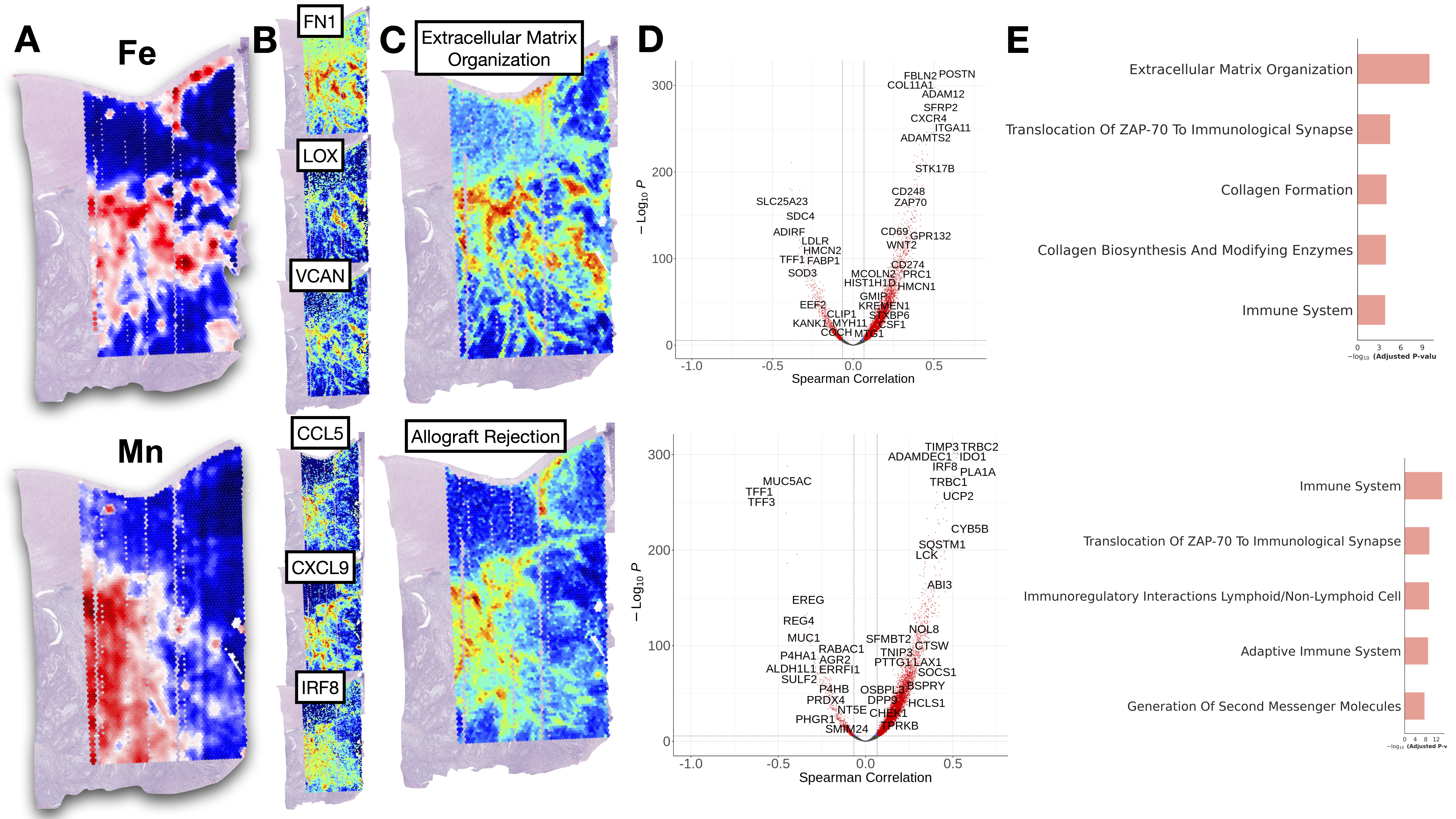

Supplement: mfaf034_Supplemental_Files [file mfaf034_supplemental_files.zip › s1.jpg]

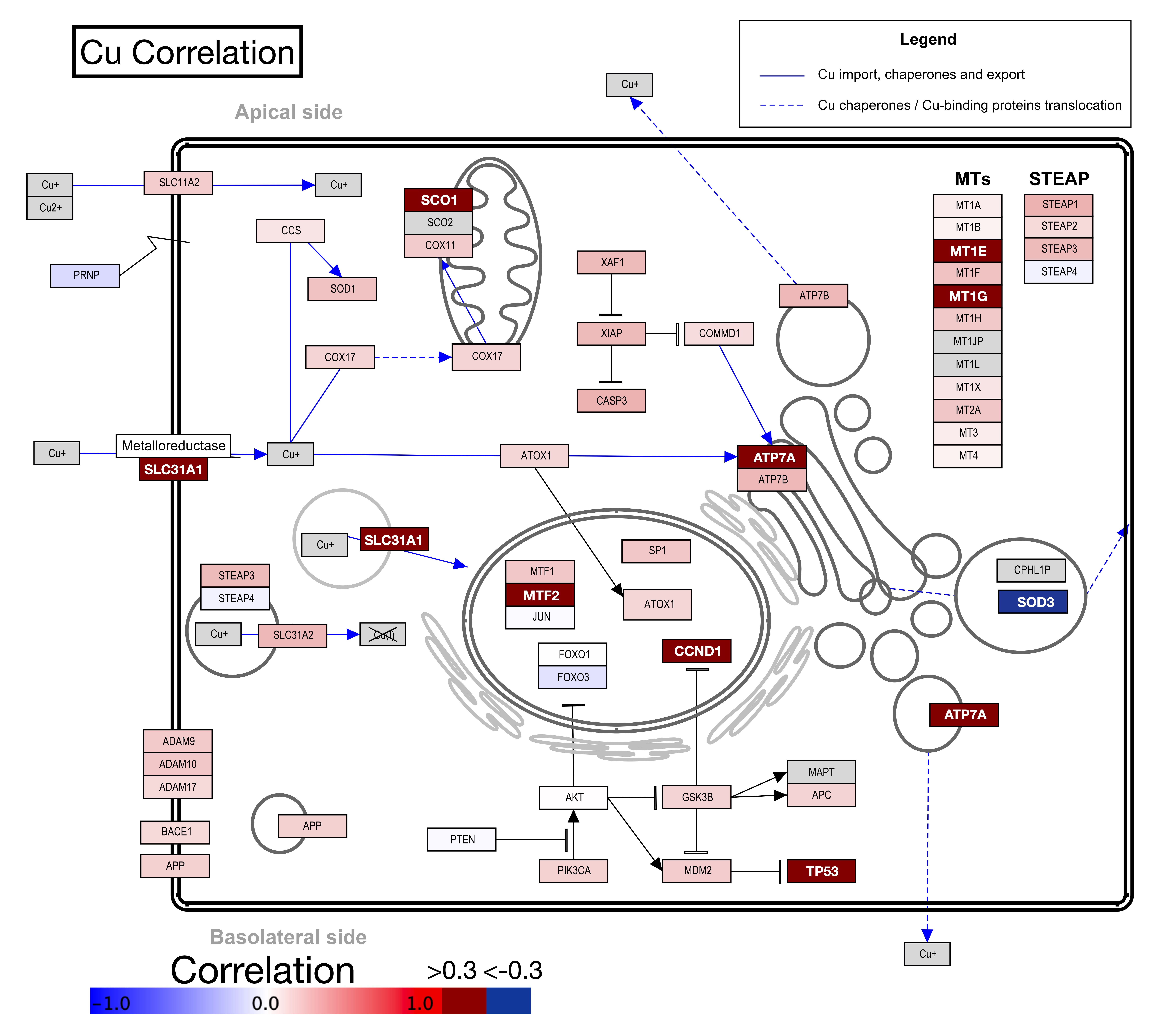

Supplement: mfaf034_Supplemental_Files [file mfaf034_supplemental_files.zip › s2.jpg]

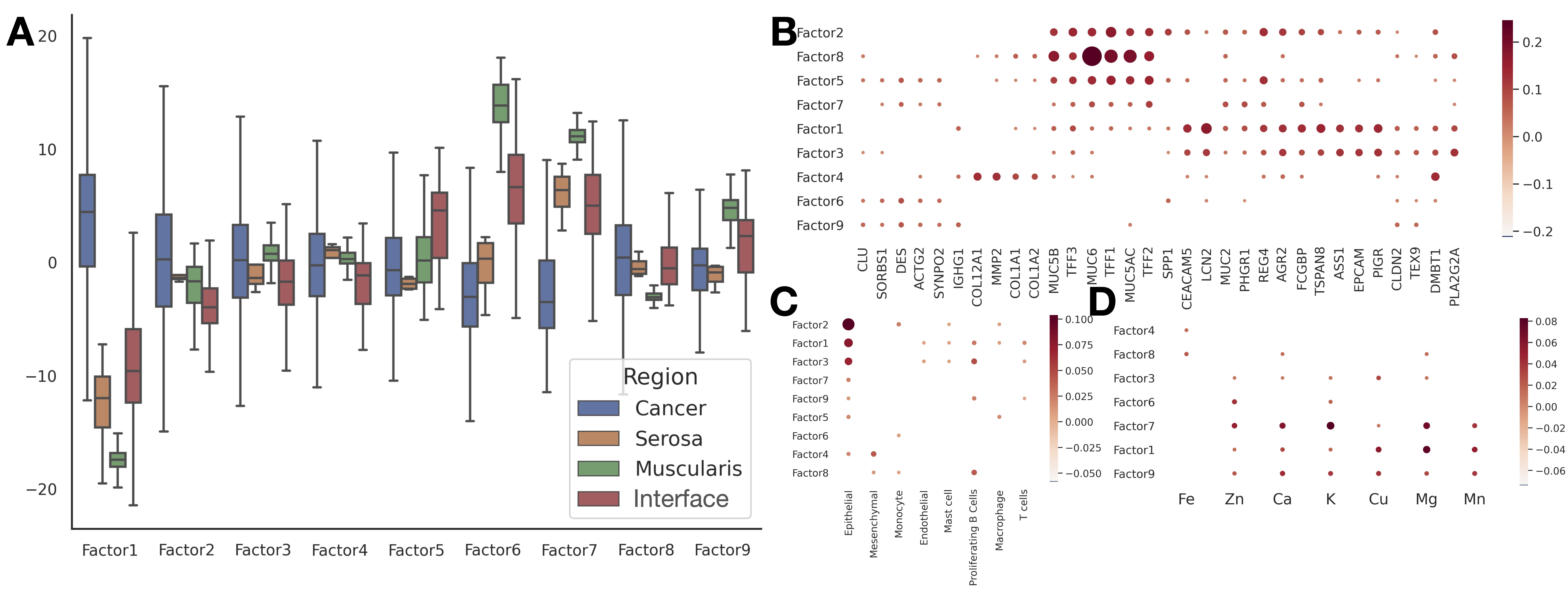

Supplement: mfaf034_Supplemental_Files [file mfaf034_supplemental_files.zip › s4.jpg]

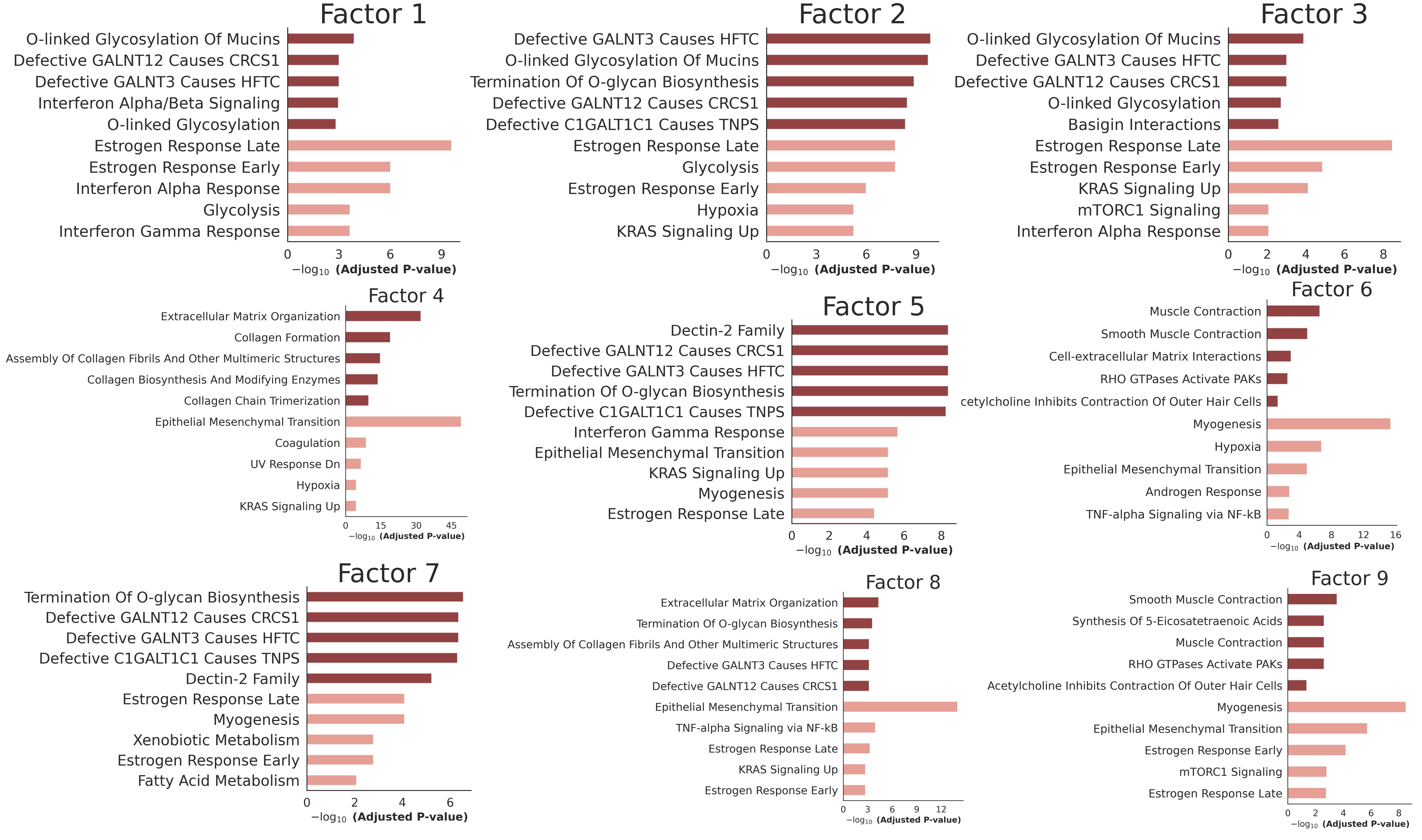

Supplement: mfaf034_Supplemental_Files [file mfaf034_supplemental_files.zip › s5.jpg]

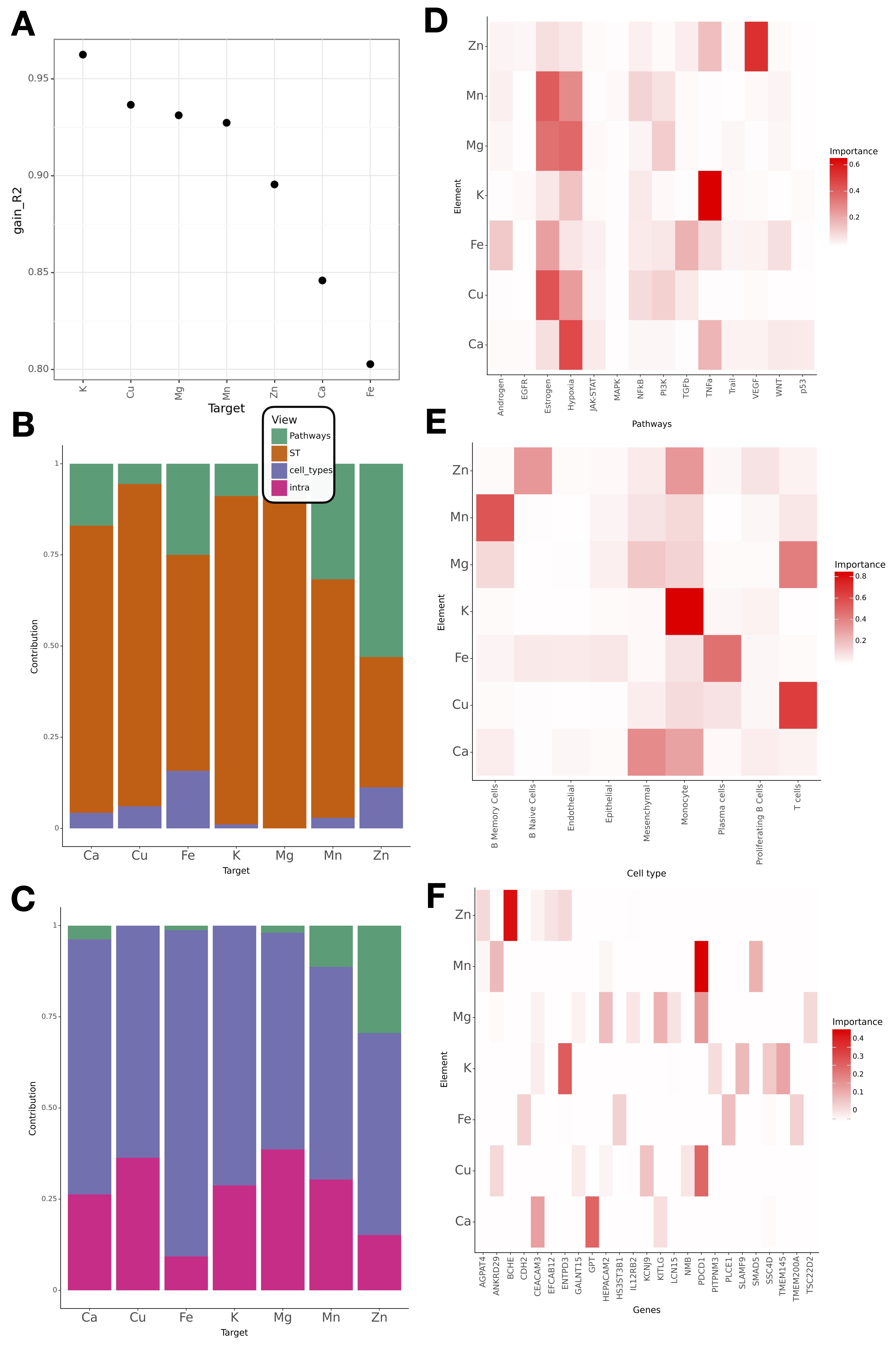

Supplement: mfaf034_Supplemental_Files [file mfaf034_supplemental_files.zip › s6.jpg]

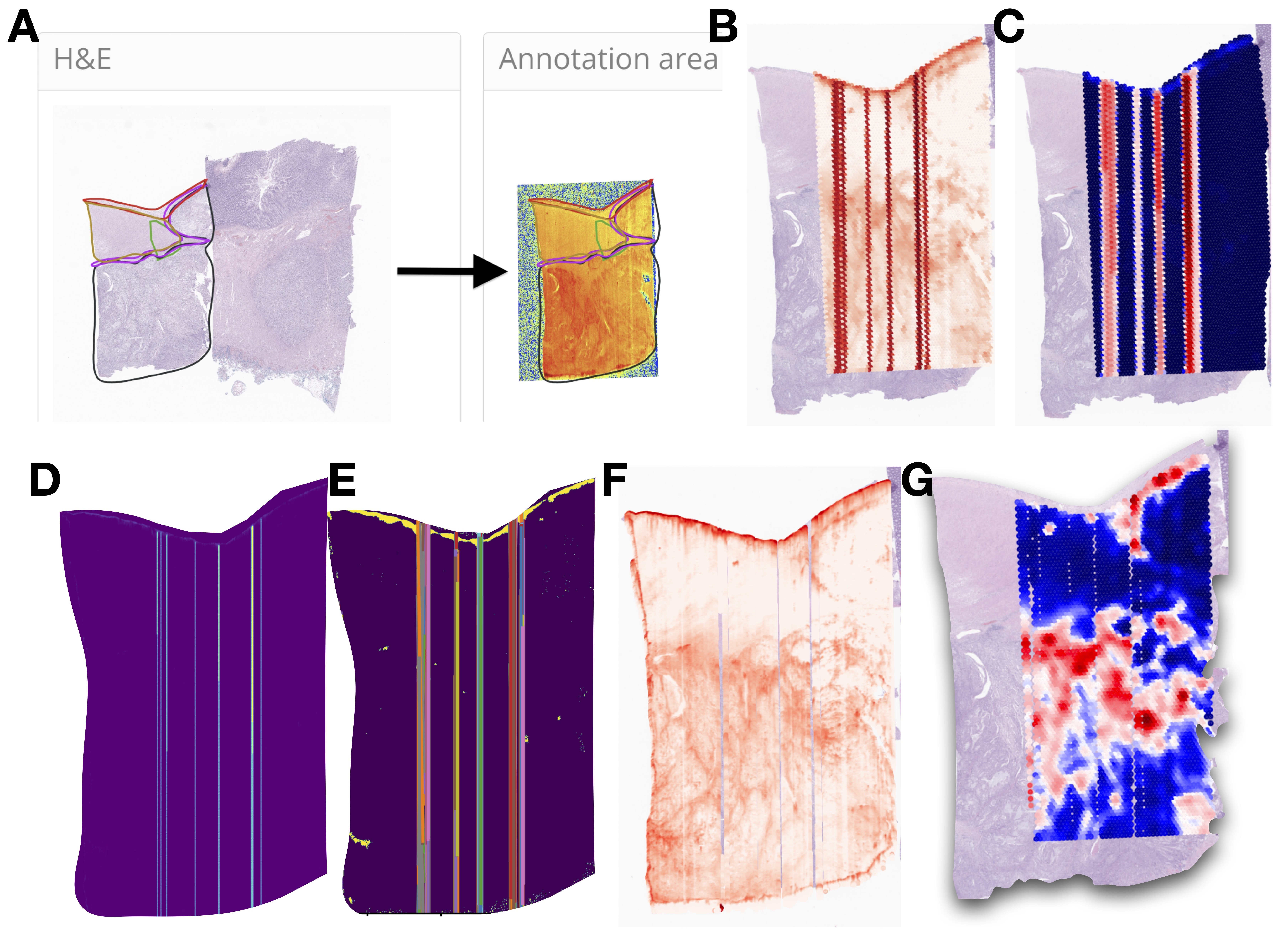

Supplement: mfaf034_Supplemental_Files [file mfaf034_supplemental_files.zip › s7.jpg]
